# Dynamics of Electron Transfers in Photosensitization Reactions of Zinc Porphyrin Derivatives

**DOI:** 10.3390/molecules28010327

**Published:** 2022-12-31

**Authors:** Soohwan Kim, Taesoo Kim, Sunghan Choi, Ho-Jin Son, Sang Ook Kang, Jae Yoon Shin

**Affiliations:** Department of Advanced Materials Chemistry, Korea University, Sejong 30019, Republic of Korea

**Keywords:** Zn porphyrin, TiO_2_, electron transfer, photosensitizer, time-resolved spectroscopy

## Abstract

Photocatalytic systems for CO_2_ reduction operate via complicated multi-electron transfer (ET) processes. A complete understanding of these ET dynamics can be challenging but is key to improving the efficiency of CO_2_ conversion. Here, we report the ET dynamics of a series of zinc porphyrin derivatives (**ZnPs**) in the photosensitization reactions where sequential ET reactions of **ZnPs** occur with a sacrificial electron donor (SED) and then with TiO_2_. We employed picosecond time-resolved fluorescence spectroscopy and femtosecond transient absorption (TA) measurement to investigate the fast ET dynamics concealed in the steady-state or slow time-resolved measurements. As a result, Stern-Volmer analysis of fluorescence lifetimes evidenced that the reaction of photoexcited **ZnPs** with SED involves static and dynamic quenching. The global fits to the TA spectra identified much faster ET dynamics on a few nanosecond-time scales in the reactions of one-electron reduced species (**ZnPs^•–^**) with TiO_2_ compared to previously measured minute-scale quenching dynamics and even diffusion rates. We propose that these dynamics report the ET dynamics of **ZnPs^•–^** formed at adjacent TiO_2_ without involving diffusion. This study highlights the importance of ultrafast time-resolved spectroscopy for elucidating the detailed ET dynamics in photosensitization reactions.

## 1. Introduction

Various photocatalytic systems, mainly consisting of photosensitizers and catalysts, have recently been developed for CO_2_ reduction [1,2,3,4]. In these systems, the photosensitizer efficiently absorbs visible light and generates electrons that can transfer to the catalyst as an initial step of the photocatalytic process. The catalyst then converts CO_2_ into energy-rich compounds, utilizing the transferred electrons. Intra- or intermolecular electron transfer (ET) is a prerequisite process in photocatalytic CO_2_ reduction, and its dynamics can affect the overall performance of the photocatalytic system. Therefore, it is crucial to elucidate the detailed dynamics and mechanisms of ET in the photocatalytic reactions for developing efficient photocatalytic systems.

However, tracking the overall ET dynamics of photocatalytic systems during the CO_2_ reduction process is challenging. The complete cycle of CO_2_ reduction requires sacrificial electron donors (SEDs) and other additives that can aid CO_2_ binding to the catalyst [5,6,7,8]. Furthermore, advanced photocatalytic systems often use metal-organic frameworks (MOFs) or semiconductors like TiO_2_ as mediators that can collect and transport multi-electrons to enhance ET efficiency and reduction performance [4,9,10,11]. Hence, the overall reactions of photocatalytic systems commonly involve multiple ET processes whose time scales span a wide range from picoseconds even to minutes, complicating the reaction dynamics and mechanisms. Deciphering the complete ET processes in photocatalytic reactions demands thorough time-resolved studies on a wide time scale under delicately controlled experimental conditions.

**Figure 1:**
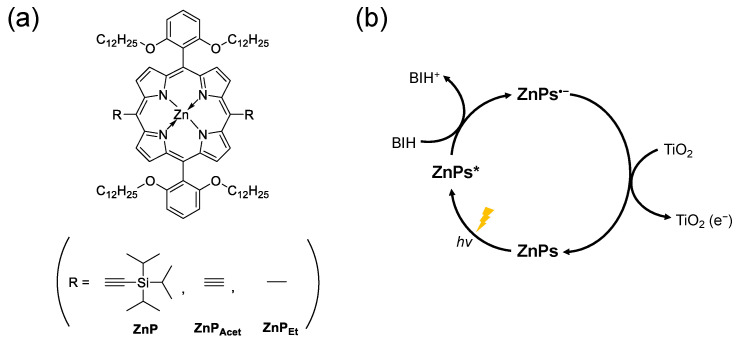
(**a**) Molecular structures of **ZnPs** and (**b**) electron pathways investigated in this study.

In this study, we investigated the ET dynamics in photosensitization reactions of a series of zinc porphyrin derivatives (**ZnPs**), which have different peripheral substituents shown in Figure 1a, using ultrafast spectroscopies, i.e., picosecond time-resolved fluorescence spectroscopy and femtosecond transient absorption (TA) measurements. Figure 1b shows the overall ET pathways tracked in this study. Our previous studies have shown that the **ZnPs** can serve as a photosensitizer in the binary hybrid system with a heterogeneous TiO_2_/Re(I) (Re(I) catalyst anchored to TiO_2_ particle) for photocatalytic CO_2_ reduction. However, the collisional ET from **ZnPs** to TiO_2_ was measured to be substantially slow (*k* = ~10^−3^ s^−1^) with high activation energy (58 kJ/mol) determined by temperature-dependent and second-to-minute-resolved kinetics of UV/vis spectrum [9]. Herein, equipped with a high time resolution, we revisited the ET reactions of **ZnPs** with a SED and TiO_2_ particle to unveil the fast dynamics possibly occurring in the photosensitization reactions. Stern-Volmer analysis of the fluorescence lifetimes yielded quenching rates that report on the ET dynamics between photoexcited **ZnPs** (ZnPs*) and SED. Additionally, global analysis of TA spectra revealed the dynamics of further ET reaction between one electron reduced species (OERS) of **ZnPs** (**ZnPs^•–^**) and TiO_2_ particle, enabling us to estimate reaction rates that are significantly faster than those previously measured with low time resolution and even faster than the diffusion rates in solution. This suggests the ET dynamics of **ZnPs^•–^** occurring very near the TiO_2_ surface.

## 2. Results and Discussion

**Figure 2:**
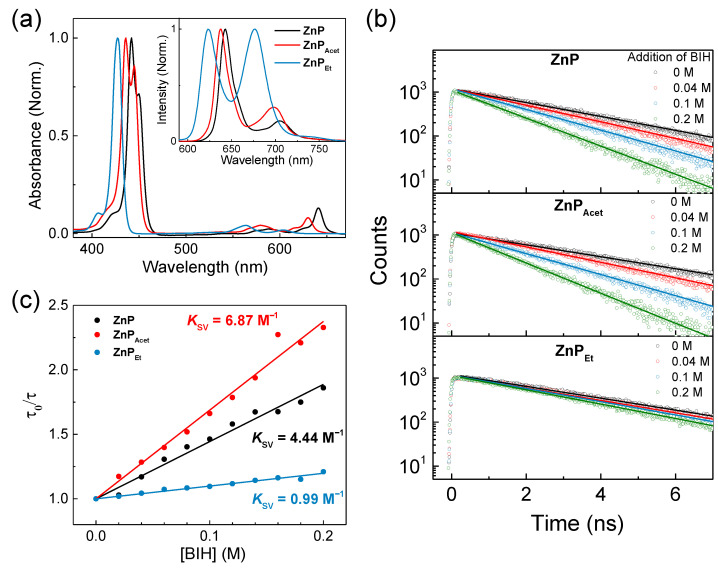
(**a**) Steady-state absorption and emission (inset) spectra of **ZnPs** in DMF, (**b**) representative fluorescence decay profiles of **ZnPs** with the addition of BIH (λ_ex_ = 400 nm; λ_det_ = 645, 635, and 620 nm for **ZnP**, **ZnP_Acet_**, **ZnP_Et_**, respectively; solid lines are the single exponential fit; see the complete set of decay profiles in Figure S1 of the Supporting Information), and (**c**) Stern-Volmer plot (solid lines are the linear fit).

Figure 2a shows the steady-state UV/vis absorption spectra of **ZnPs**. They have typical spectral features of metalloporphyrin with an intense Soret (B) band in the range of 400 to 470 nm and a Q-band in a region longer than 580 nm. The steady-state emission spectra appear as a mirror image of the Q-band (inset of Figure 2a). As the substituent changes from silanyethynyl to ethynyl and ethyl, absorption and emission spectra blue-shifts due to weakened electron-donating ability for **ZnP**→**ZnP_Acet_** and broken π-conjugation for **ZnP_Acet_**→**ZnP_Et_**. The fluorescence lifetimes of **ZnP_Acet_** and **ZnP_Et_** are almost identical, having a time constant of 2.9 and 3.0 ns, respectively (Table 1). However, **ZnP** has a slightly shorter fluorescence lifetime with a time constant of 2.4 ns (Table 1): its bulky substituent, silanyethynyl, can supply more nonradiative decay channels, presumably via vibrational relaxation, enhancing the fluorescence decay rate. Here, we did not observe any hint of aggregation under the sample concentration (10 μM) in the UV/VIS absorption spectra, and thus, the measured fluorescence lifetimes were not affected by aggregates in **ZnPs**.

In the photosensitization reactions of **ZnPs**, the first step is the ET reaction of photoexcited **ZnPs** with BIH to form an OERS, **ZnPs^•–^**. BIH plays a role as a SED in this reaction. Then, an excess electron of **ZnPs^•–^** is transferred to TiO_2_, which serves as an electron reservoir for ET to the Re(I) catalyst in the binary hybrid system, when the OERS encounters TiO_2_ particles. To understand the overall dynamics of this reductive quenching mechanism, we first measured fluorescence quenching kinetics of **ZnPs** with BIH, i.e., the dynamics of **ZnPs^•–^** formation, as displayed in Figure 2b. The fluorescence lifetimes of **ZnPs** decrease with the addition of BIH because collisional ET with BIH quenches the fluorescence. The higher concentrations of BIH, the higher collisional frequency, and thus, the shorter lifetimes of **ZnPs**. The relationship between the fluorescence lifetimes and the concentration of a quencher, [Q], is described by the Stern-Volmer equation: [12]

(1) $$\frac{\tau_{0}}{\tau }=1+K_{\mathrm{SV}}\left[Q\right]$$

where *τ*_0_ and *τ* are the fluorescence lifetimes in the absence and presence of quencher, respectively. *K*_SV_ is the Stern-Volmer quenching constant and is given by *K*_SV_ = *k*_q_*τ*_0_ where *k*_q_ is the bimolecular quenching constant. According to Equation (1), the linear fits to the Stern-Volmer plot of the fluorescence lifetimes resulted in *K*_SV_ values, and corresponding *k*_q_ values were calculated from *τ*_0_ (Figure 2c and Table 1). The calculated *k*_q_ values of **ZnP** and **ZnP_Acet_** are 6 to 8-fold larger than that of **ZnP_Et_**, which is consistent with the driving force (ΔG) of ET from BIH to photoexcited **ZnPs**: based on the oxidation and reduction potentials in the cyclic voltammetry measurements the estimated ΔG is −0.34/−0.36 eV for **ZnP**/**ZnP_Acet_** and −0.10 eV for **ZnP_Et_** [9]. Unlike conjugated substituents in **ZnP** and **ZnP_Acet_**, non-conjugated substituent, ethyl, in **ZnP_Et_** does not lower the molecular orbital (MO) energy levels, keeping the reduction potential of photoexcited **ZnP_Et_** close to the oxidation potential of BIH and yielding less negative ΔG. More negative ΔG generally coincides with a lower reaction barrier and, thus, a faster reaction rate as we observed larger *k*_q_ values for **ZnP** and **ZnP_Acet_**.

Interestingly, the *k*_q_ value of **ZnP** obtained by the fluorescence lifetime measurements in this study is much smaller than the previously acquired value by quenching the fluorescence intensity (*k*_q_ [10^9^ M^−1^ s^−1^] = 1.9 vs. 9.3). Different origins of quenching mechanisms can explain the discrepancy in the quenching rates between two methods. The quenching rate determined by the fluorescence lifetime measurements only reports on the collisional (or dynamic) quenching. In contrast, the method with the fluorescence intensity can include the quenching through the formation of a nonfluorescent ground-state complex between fluorophore and quencher (static quenching) as well [12]. If both dynamic and static quenching happens, the fluorescence intensity measurements will result in larger *k*_q_ values than those from the fluorescence lifetime measurements even for the same quenching reaction. Therefore, the discrepancy in **ZnP**’s *k*_q_ observed by two methods suggests the possibility of complex formation between **ZnP** and BIH in the ground state. In many cases, transition metals form complexes with imidazole and benzimidazole derivatives in porphyrins and phthalocyanines [13,14,15].

On the other hand, the difference in *k*_q_ values between the two methods is not significant for **ZnP_Acet_**. (*k*_q_ determined by the fluorescence intensity quenching is 3.0 × 10^9^ M^−1^ s^−1^) [9]. The electron-donating from the substituent is less in **ZnP_Acet_** than in **ZnP**, which can reduce the complex formation between **ZnP_Acet_** and BIH. For **ZnP_Et_**, the fluorescence quenching by BIH was not significant in both intensity and lifetime measurements, and it seems unreliable to compare the values and discuss the complex formation.

**Figure 3:**
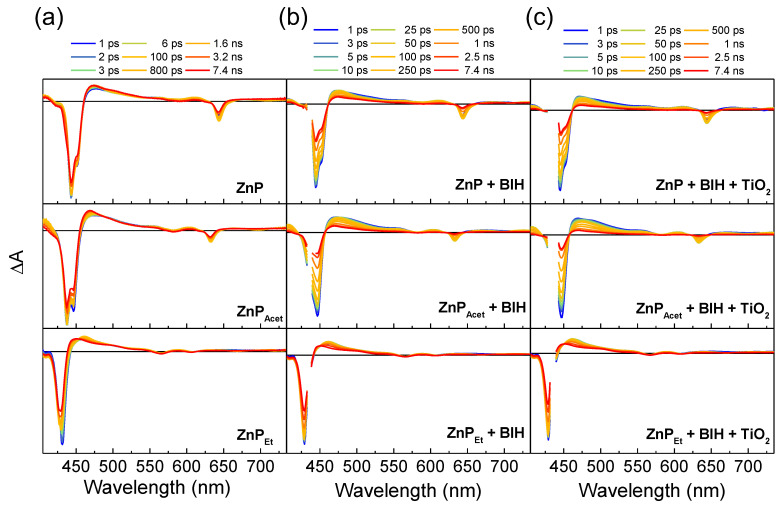
TA spectra at selected time delays of (**a**) **ZnPs**, (**b**) **ZnPs** + BIH, and (**c**) **ZnPs** + BIH + TiO_2_ in DMF. The amount of each component in the 4 mL solution was 10 μM **ZnPs**, 0.3 M BIH, and 1 mg TiO_2_. The data near 435 nm were omitted in (**b**,**c**) due to the scattered pump light.

To investigate the ET dynamics of the further photosensitization reaction beyond the formation of **ZnPs^•–^**, we carried out femtosecond TA measurements initially with **ZnPs** and then in the presence of BIH or/and TiO_2_ as depicted in Figure 3 (see also Figure S2 in Supporting Information). In the absence of BIH and TiO_2_, the TA spectra of **ZnPs** do not show dramatic changes within the time delays of 7.4 ns (Figure 3a). For **ZnPs** and **ZnP_Acet_**, the ground-state bleach signals of the Soret band near 430~450 nm and the Q-band near 630~640 nm slightly recover, concurring with modest growth of induced absorption signals near 475 nm. The TA spectra of **ZnP_Et_** also exhibit similar behaviors except for minor decay of induced absorption signal near 460 nm. In the presence of BIH, the TA spectra of **ZnP** and **ZnP_Acet_** substantially evolve with time (Figure 3b): both the ground-state bleach and induced absorption signals significantly decrease in magnitude with an increase of time delay, reflecting the ET reaction with BIH. (Note that the recovery of the ground-state bleach signal represents a mixed evolution of the ground-state recovery of ZnPs* and the absorption of newly formed **ZnPs^•–^**.) However, we could not observe any spectral feature attributable to **ZnPs^•–^** presumably due to the overlaps of TA signals and the limited range of our probe wavelength. For the TA spectra of **ZnP_Et_**, the presence of BIH does not seem to primarily affect their evolution in line with the minute change of fluorescence lifetimes observed in the quenching measurements. Finally, adding TiO_2_ into the sample solution in the presence of BIH enhances the decrease of bleach and absorption signals in the TA spectra of **ZnP** and **ZnP_Acet_**, which implies the ET dynamics from **ZnPs^•–^** to TiO_2_ particle. In contrast, the TA spectra of **ZnP_Et_** measured with the addition of BIH and TiO_2_ reveal the same behaviors as those with only BIH added.

**Figure 4:**
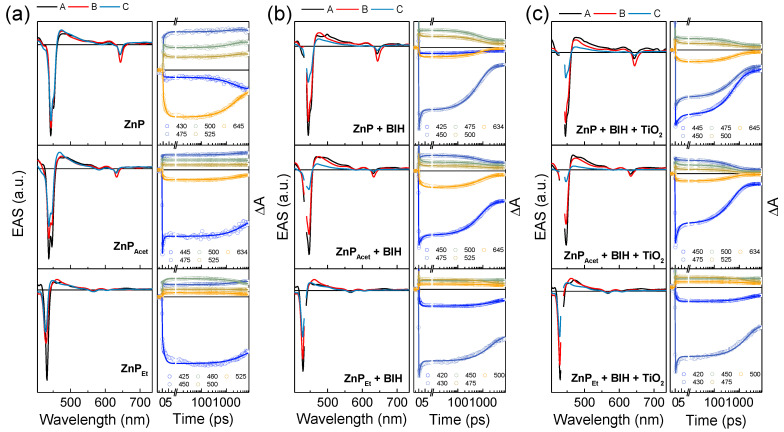
EAS (left) and representative decay profiles (right) of (**a**) **ZnPs**, (**b**) **ZnPs** + BIH, and (**c**) **ZnPs** + BIH + TiO_2_ in DMF obtained from the global fits to the TA data. The first-order kinetic model (A→B→C→ground state (GS)) was used in the fit. The EAS data near 435 nm were omitted in (**b**,**c**) due to the scattered pump light. The solid lines in the decay profiles are the fits.

To analyze the evolution of TA spectra in detail, we globally fitted the TA data with the first-order kinetic model. The kinetic model for the best fit to all the TA data required three species, A→B→C→ground state (GS). Figure 4 displays evolution-associated spectra (EAS) obtained from the fits and representative decay profiles. The kinetic parameters associated with each EAS are tabulated in Table 2 as an inverse form of the time constant. Without any additives, the TA spectra of **ZnPs** can be fitted with the associated time constants (*τ*) of 0.7~2.2 ps for A→B, 2.3~3.4 ns for B→C, and more than 100 ns for C→GS. The first evolution, A→B, in a few picoseconds is ascribed to the internal conversion (IC) process from the higher singlet excited-state (S_n_) to the lowest singlet excited-state (S_1_) since the pump wavelength was tuned to the B-band excitation at 435 nm in the TA measurements [16,17,18,19]. Then, the B state of EAS, which corresponds to the S_1_ state, evolves into the C state with *τ*_B→C_ that matches the singlet state lifetime (*τ*_0_) measured with the time-resolved fluorescence, enabling us to assign the C state of EAS as the triplet excited state (T_1_) of **ZnPs**. The C state lives longer than the upper limit of our apparatus’s time delay (~8 ns) and has a long time constant (~100 ns) from the global fit, confirming its triplet character.

Even with the addition of BIH or/and TiO_2_, the EAS of A, B, and C states do not essentially change from those observed in **ZnPs**, still representing the S_n_, S_1_, and T_1_ states, respectively: their *τ*_A→B_ values of 1.1~2.8 ps are the same as the IC process (S_n_→S_1_) in **ZnPs**, and *τ*_C→GS_ values of longer than 100 ns are agreeable to the triplet state decay (Figure 4 and Table 2). However, the presence of additives in the **ZnPs** solution mainly affects the S_1_ state lifetime, *τ*_B→C_. Like in the fluorescence quenching experiments where the ET reaction with BIH shortens the S_1_ state lifetime of **ZnPs**, *τ*_B→C_ decreases in the TA measurements with the addition of 0.3 M BIH: the fitted values of *τ*_B→C_ are 822 and 737 ps for **ZnP** and **ZnP_Acet_** and 2.0 ns for **ZnP_Et_** (Table 2). These *τ*_B→C_ values are comparable to the S_1_ state lifetimes for **ZnPs** + 0.3 M BIH (*τ***_ZnPs_**_+BIH_), which are estimated to be 1.0, 0.9, and 2.4 ns for **ZnP**, **ZnP_Acet_**, and **ZnP_Et_**, respectively, by

(2) $$\frac{1}{\tau_{\mathrm{ZnPs}+\mathrm{BIH}}}=\frac{1}{\tau_{0}}+0.3k_{q}$$

(where *τ*_0_ and *k*_q_ are from Table 1 and 0.3 is from the concentration of BIH). This demonstrates that the TA experiments reveal the ET dynamics of **ZnPs** with BIH, which is consistent with the results from the Stern-Volmer analysis.

In **ZnP** and **ZnP_Acet_**, the *τ*_B→C_ value further decreases with the addition of both BIH and TiO_2_ (Table 2), meaning that the decay of the S_1_ state is additionally enhanced by the presence of TiO_2_ in the solution. According to the reductive quenching mechanism, the electron transfer to TiO_2_ should occur not directly from the photoexcited **ZnPs** in the S_1_ state (ZnPs*) but from the OERS (**ZnPs^•–^**) that is formed after the ET reaction with BIH. Indeed, the TA experiments with only TiO_2_ added to the **ZnPs** solution showed no change in the kinetics of TA spectra compared to the sample solution without additives (Figure S2 and Table S1), confirming that the direct reaction between ZnPs* and TiO_2_ is unlikely. However, the TA spectra of **ZnPs^•–^** can not be resolved in our experiments, and tracking its explicit dynamics is impossible. Instead, the disappearance of **ZnPs^•–^** by the ET reaction with TiO_2_ will be incorporated in the decay of ZnPs* because quenching of **ZnPs^•–^** by TiO_2_ generates the ground state species (**ZnPs**) that has no TA signal, conceivably contributing to the decay of ZnPs*, i.e., the additional enhancement of decay rate in the B→C evolution. Assuming that quenching by TiO_2_ is the only factor for the *τ*_B→C_ decrease, we can estimate the quenching rate of **ZnPs^•–^** in the ET reaction with TiO_2_ (*k*_q,OERS_) as

(3) $$k_{q,\;\mathrm{OERS}}=\frac{1}{\tau_{B\to C,\;\mathrm{BIH}+{\mathrm{TiO}}_{2}}}-\frac{1}{\tau_{B\to C,\;\mathrm{BIH}}}$$

where *τ*_B→C,BIH_ and *τ*_B→C,BIH+TiO2_ are the time constants for the B→C evolution measured in the presence of BIH and BIH + TiO_2_, respectively (Table 2). The calculated *k*_q,OERS_ values of **ZnP** and **ZnP_Acet_** are $4.12\times 10^{8}$ and $5.12\times 10^{8}$ s^−1^, respectively. In **ZnP_Et_**, the formation of OERS is so slow that its quenching by TiO_2_ is not observed within the experimental time window of TA measurements, i.e., no change in *τ*_B→C_ with the addition of TiO_2_. Compared to the collisional ET rates of **ZnP^•–^** with TiO_2_ previously determined by the UV/vis spectrum change ($5.12\times 10^{-3}$ s^−1^), [9] the *k*_q,OERS_ values of **ZnP** and **ZnP_Acet_** differ by many orders of magnitude. Given that the concentration of BIH was lower only by three times in the UV/vis spectrum measurements with the same concentration of TiO_2_, the fast ET dynamics of the OERS observed in this study should reflect a different mechanism from the high activation energy process formerly revealed with a slow time resolution, suggesting another ET route.

In fact, the ET dynamics of the OERS happening on a few nanosecond time scales are even faster than the diffusion rate in solution. The diffusion rate constant of a dye in most organic solvents is typically on the order of 10^9^~10^10^ M^−1^ S^−1^ at room temperature [20]. Under our experimental condition with 10 μM of **ZnPs**, the concentration of the OERS formed after the ET between photoexcited **ZnPs** and BIH will be much less than 10 μM. Then, the diffusion rate constant of the OERS will become far less than 10^4^ s^−1^ which is significantly slower than the *k*_q,OERS_ values obtained here. (Note that the particle size of TiO_2_ used in this study spans from 0.2~3 μm, [21], i.e., the particles are very large, and we can assume that their diffusion is almost negligible compared to **ZnPs**). In this regard, the *k*_q,OERS_ values may report the ET dynamics of the OERS formed at the very vicinity of the TiO_2_ surface. The interfacial electron transfer on the semiconductor surface generally occurs on picoseconds time scale or even faster times [22,23,24,25,26,27,28] KC et al. reported that when the **ZnP** derivative is covalently attached to the TiO_2_ surface, the electron injection from **ZnP^•–^** to TiO_2_ occurs within 30 ps [29]. Therefore, a few nanoseconds dynamics observed in this study can correspond to the ET dynamics of intermediate regime where the dye molecules in solution are very near the semiconductor surface without a direct connection like a covalent bond and electronic interaction between the dye and surface is negligible. Still, no diffusion of dye is required for the ET in this regime. This implies that the overall intermolecular ET process in photosensitization reaction can happen on multi-time scales, and thus, the spectroscopies with the time resolution of multi-time scales are required to uncover the complete intermolecular ET dynamics.

## 3. Materials and Methods

All reagents were purchased from Sigma Aldrich (St. Louis, MO, USA) and used without further purification. TiO_2_ (Hombikat UV-100) was purchased from Huntsman (The Woodlands, TX, USA). *N*,*N*-Dimethylformamide (DMF) was distilled from calcium hydride and stored over molecular sieves. [5,15-bis[(triprop-2-yl silanyl)ethynyl]-10,20-bis[2,6-di(dodecyloxy)phenyl]porphinato]zinc (**ZnP**), [5,15-diethynyl-10,20-bis[2,6-di(dodecyloxy)phenyl]porphinato]zinc (**ZnP_Acet_**), and [5,15-diethyl-10,20-bis[2,6-di(dodecyloxy)phenyl]porphinato]zinc (**ZnP_Et_**) samples and 1,3-dimethyl-2-phenyl-1,3-dihydrobenzimidazole (BIH) were synthesized according to the previously reported procedures [9,10].

Steady-state absorption and emission spectra were collected using a Cary 5000 UV-Vis-NIR (Agilent Technologies, Inc., Santa Clara, CA, USA) and Cary Eclipse (Varian, Palo Alto, CA, USA), respectively. For picosecond time-resolved fluorescence spectroscopy, a home-built cavity-dumped Kerr-lens mode-locked Ti:sapphire oscillator was used. The 800 nm output was doubled in frequency using a 100 μm thick BBO (β-barium borate) crystal to generate the excitation pulses at 400 nm. A parabolic mirror was employed to focus the excitation pulse onto the sample and to collect fluorescence with a confocal geometry. The collected light was sent to a monochromator (SP-2155, Teledyne Princeton Instruments, Thousand Oaks, CA, USA) and detected with a single-photon counting module (id 100-50, id Quantique). A commercial TCSPC board (SPC-130-EMN, Becker & Hickl Inc., Berlin, Germany) was used to record time-resolved fluorescence with a time resolution of about 50 ps. All the instruments were controlled in unison by using a home-built LabVIEW software (LabVIEW 2016, 16.0, 32 bit, National Instruments, Austin, TX, USA).

Femtosecond TA measurements were previously described in detail [30]. Briefly, a Ti:sapphire regenerative amplifier system at 1 kHz (Spitfire Ace, Spectra Physics, Inc., Milpitas, CA, USA), which was seeded by a Ti:sapphire oscillator (MaiTai SP, Spectra Physics, Inc., Milpitas, CA, USA) and pumped by a diode-pumped Q-switched laser (Empower, Spectra Physics, Inc., Milpitas, CA, USA), was used for time-resolved measurements. An optical parametric amplifier (TOPAS prime, Spectra Physics, Inc., Milpitas, CA, USA) converted the 800 nm fundamental output into 435 nm for excitation. For TA measurements, a small residual of 800 nm fundamental light was focused on a water-filled cuvette to generate white light probe pulses directed to the computer-controlled translational delay stage. The mechanical chopper alternatively blocked pump pulses at 500 Hz to calculate TA spectra from the probe intensity detected by the CCD detector when the pump is on and off. Both pump and probe pulses were focused on a 1 mm-sandwich sample cell containing a magnetic stirring bar. The TA spectra were recorded by a commercial pump-probe spectrometer (Helios, Ultrafast Systems, LLC, Sarasota, FL, USA). Before kinetic analysis, the TA data were background-subtracted and chirp-corrected by using Surface Xplorer 4 (Ultrafast Systems, LLC, Sarasota, FL, USA). Kinetic data from multiple different wavelengths fit a first-order kinetic model using the global analysis programs written in MATLAB.

## 4. Conclusions

In conclusion, we investigated the ET dynamics of **ZnPs** during the photosensitization reaction where ZnPs* react with BIH, forming **ZnPs^•–^** that can transfer an excess electron to TiO_2_. Here, the time-resolved emission and absorption spectroscopies uncovered more detailed dynamics and mechanisms in the ET reactions compared to the previously reported steady-state measurements. The Stern-Volmer analysis of the fluorescence lifetime quenching experiments revealed a large discrepancy in *k*_q_ of **ZnP** between the steady-state and time-resolved measurements, suggesting that both static and dynamic quenching processes exist in the reaction of **ZnP** and BIH. Most of all, the TA measurements unveiled the fast ET dynamics between the OERS and TiO_2_, which occurs on a time scale of a few nanoseconds. This fast time scale is even faster than the diffusion rate in solution, suggesting that diffusion is not involved in this ET reaction. We propose that the ET from the OERS formed at the very vicinity of the TiO_2_ surface is responsible for the fast time scale ET dynamics.

Designing more efficient photosensitizers for photocatalytic CO_2_ reduction requires a thorough understanding of the ET mechanisms in the photosensitization reaction. This study demonstrates that the ET dynamics of photosensitizers can span multi-time scales and highlights the importance of time-resolved spectroscopies with multi-scale time resolution for elucidating the reaction mechanisms of photosensitization.

## Figures and Tables

**Table 1:** Quenching Kinetic Parameters of **ZnPs** by BIH.

	*τ*_0_ (ns) ^1^	*K*_SV_ (M^−1^) ^2^	*k*_q_ (10^9^ M^−1^ s^−1^) ^3^
**ZnP**	2.4	4.44	1.9
**ZnP_Acet_**	2.9	6.87	2.4
**ZnP_Et_**	3.0	0.99	0.3

^1^ Fluorescence lifetimes measured in the absence of BIH (λ_ex_ = 400 nm; λ_det_ = 645, 635, and 620 nm for **ZnP**, **ZnP_Acet_**, **ZnP_Et_**, respectively). ^2^ Stern-Volmer quenching constant. ^3^ bimolecular quenching constant.

**Table 2:** Kinetic Parameters from the Global Fits of the TA Spectra of **ZnPs**.

	Additives	*k*_A→B_	*k*_B→C_	*k*_C→GS_
**ZnP**		(2.2 ± 0.1 ps)^−1^	(2.3 ± 0.1 ns)^−1^	<<(100 ns)^−1^
	+BIH	(2.2 ± 0.2 ps)^−1^	(822 ± 21 ps)^−1^	
	+BIH + TiO_2_	(2.8 ± 0.3 ps)^−1^	(614 ± 15 ps)^−1^	
**ZnP_Acet_**		(0.7 ± 0.1 ps)^−1^	(3.4 ± 0.2 ns)^−1^	
	+BIH	(1.1 ± 0.1 ps)^−1^	(737 ± 8 ps)^−1^	
	+BIH + TiO_2_	(2.0 ± 0.1 ps)^−1^	(535 ± 5 ps)^−1^	
**ZnP_Et_**		(1.6 ± 0.1 ps)^−1^	(2.9 ± 0.3 ns)^−1^	
	+BIH	(1.1 ± 0.1 ps)^−1^	(2.0 ± 0.1 ns)^−1^	
	+BIH + TiO_2_	(1.2 ± 0.1 ps)^−1^	(2.0 ± 0.1 ns)^−1^	

## Data Availability

Not applicable.

## Supplementary Materials

The following supporting information can be downloaded at: https://www.mdpi.com/article/10.3390/molecules28010327/s1, Figure S1: complete set of fluorescence decay profiles; Figure S2: TA data for **ZnPs** + TiO_2_; Table S1: Kinetic parameters from the global fits of **ZnPs** + TiO_2_; Figure S3: Model populations obtained from the global fits.

## Sample Availability

Samples of the compounds are not available from the authors.
