# Supplementary material for: Dynamics of Electron Transfers in Photosensitization Reactions of Zinc Porphyrin Derivatives
Source: Molecules. 2022 Dec 31;28(1):327. doi: 10.3390/molecules28010327 (PMC9822303; doi:10.3390/molecules28010327)
Supplement: Supplementary file 1 [file molecules-28-00327-s001.zip › molecules-1984923-supplementary.pdf]

Supplementary Materials for:

Dynamics of Electron Transfers in Photosensitization Reactions of Zinc Porphyrin Derivatives

Soohwan Kim, Taesoo Kim, Sunghan Choi, Ho-Jin Son, Sang Ook Kang,

and Jae Yoon Shin *

Department of Advanced Materials Chemistry, Korea University, Sejong 30019, Republic of Korea

* Correspondence: jaeyoonshin@korea.ac.kr

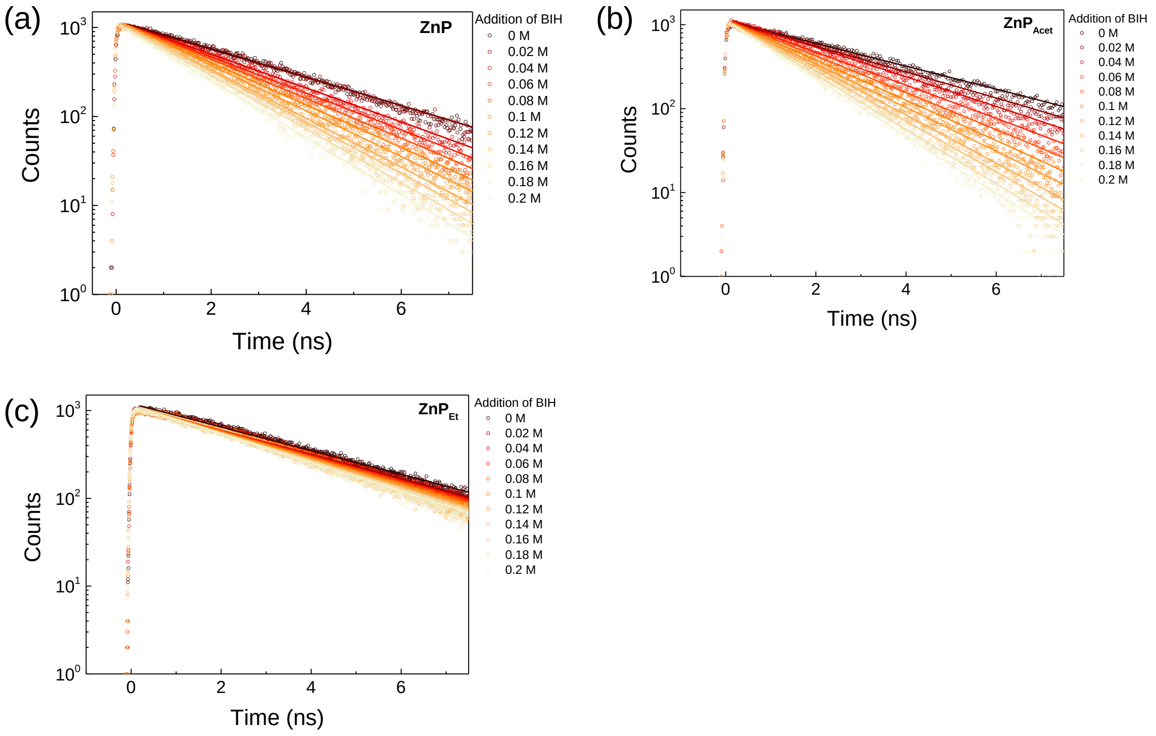

Figure S1. Fluorescence decay profiles of (a) **ZnP**, (b) **ZnP$_{Acet}$**, and (c) **ZnP$_{Et}$** with the addition of BIH (λ_{ex} = 400 nm; λ_{det} = 645, 635, and 620 nm for **ZnP**, **ZnP$_{Acet}$**, **ZnP$_{Et}$**, respectively; solid lines are the single exponential fit.).

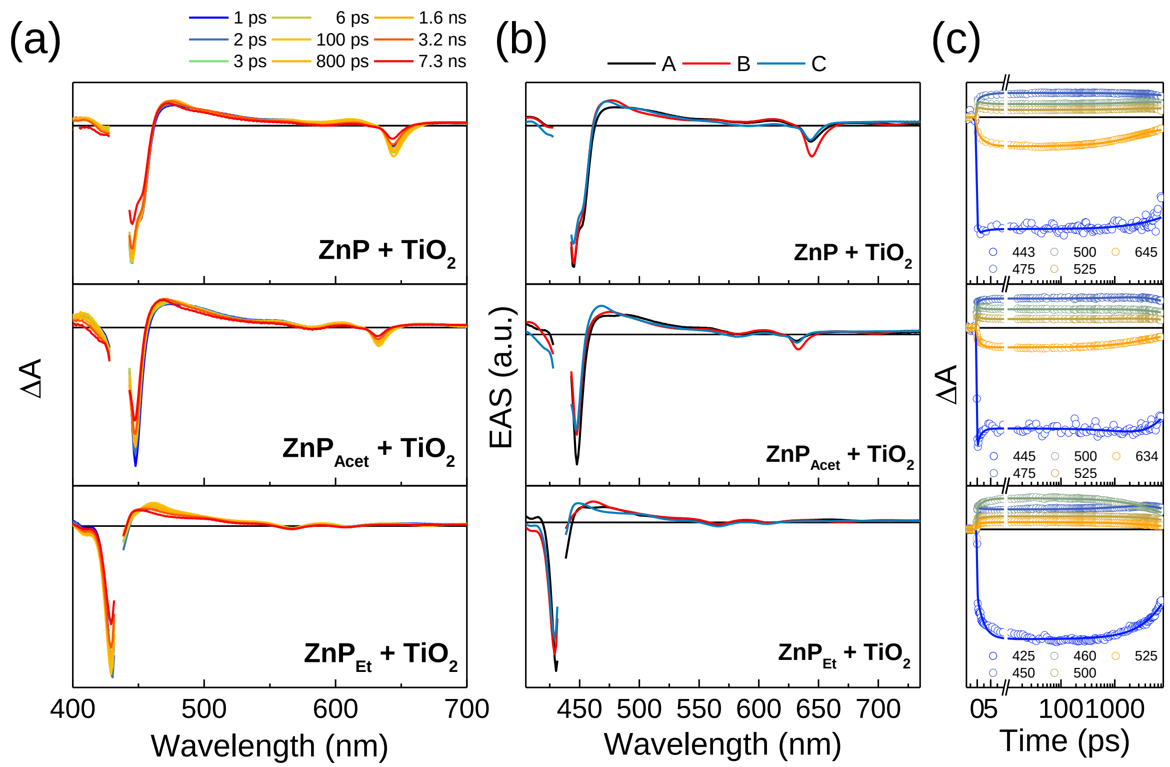

Figure S2. TA data for **ZnPs** + TiO_2: (a) TA spectra at selected time delays, (b) EAS obtained from the global fits, and (c) representative decay profiles (open circles) with fits (solid lines). The data near 435 nm were omitted in (b) and (c) due to the scattered pump light.

Table S1. Kinetic Parameters from the Global Fits of the TA Spectra of **ZnPs** + TiO_2.

	$k_{A\rightarrow B}$	$k_{B\rightarrow C}$	$k_{C\rightarrow GS}$
**ZnP** + TiO_2	$(2.7 \pm 0.5\ ps)^{-1}$	$(2.6 \pm 0.3\ ns)^{-1}$	
**ZnP**$_{Acet}$ + TiO_2	$(1.3 \pm 0.1\ ps)^{-1}$	$(3.3 \pm 0.3\ ns)^{-1}$	<< $(100\ ns)^{-1}$
**ZnP**$_{Et}$ + TiO_2	$(2.5 \pm 0.1\ ps)^{-1}$	$(3.0 \pm 0.4\ ns)^{-1}$	

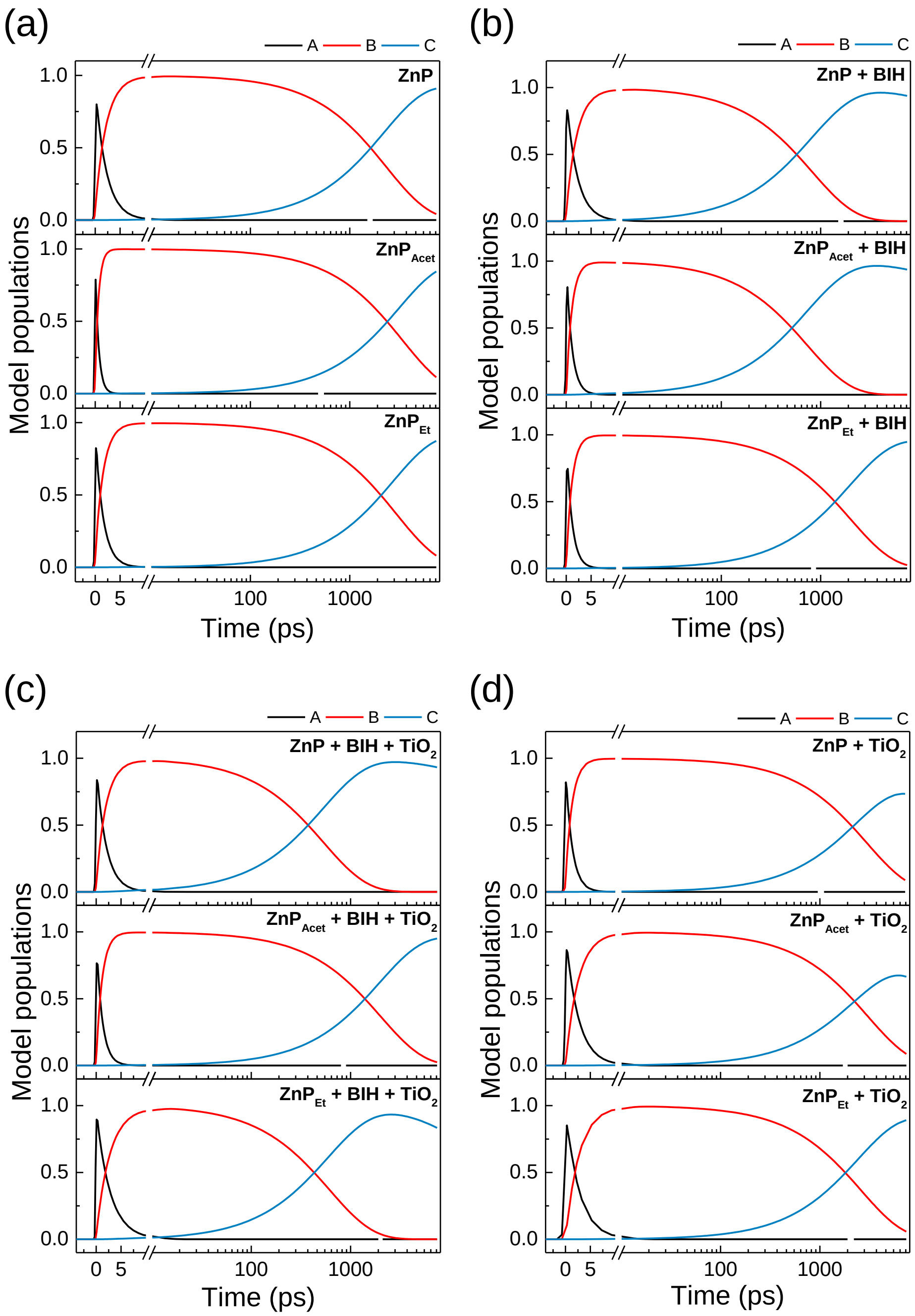

Figure S3. Model populations obtained from the global fits to TA data of (a) **ZnPs**, (b) **ZnPs** + BIH, (c) **ZnPs** + BIH + TiO_2, and (d) **ZnPs** + TiO_2.
